# What determines the success and failure of environmental crowdfunding?

**DOI:** 10.1007/s13280-021-01522-0

**Published:** 2021-03-23

**Authors:** Takahiro Kubo, Diogo Veríssimo, Shinya Uryu, Taro Mieno, Douglas MacMillan

**Affiliations:** 1 School of Anthropology and Conservation, University of Kent, 78 Marlowe Building, Canterbury, CT2 7NR UK; 2 Center for Environmental Biology and Ecosystem Studies, National Institute for Environmental Studies, Tsukuba, Japan; 3 Department of Zoology, University of Oxford, Oxford, UK; 4 Agricultural Economics, University of Nebraska-Lincoln, Lincoln, USA

**Keywords:** Behaviour, Charity, Conservation finance, Conservation marketing, Donation, Fundraising

## Abstract

Online crowdfunding can help address the perennial financial shortfalls in environmental conservation and management. Although many online crowdfunding campaigns fail to collect any funds due to not achieving their targets, little is known about what drives success. To address this knowledge gap, we applied a mixed-methods approach to data from 473 successful and failed campaigns hosted on the online crowdfunding platform Readyfor. We found that fundraising performance varied by topic, with campaigns on pet animal management outperforming those focussed on landscape management and sustainable use. We also found that marketing strategies associated with online findability and increased reach through social networks, increased fundraising success. However, the existence of other environmental campaigns running simultaneously, reduced the chance of success, which implies that the selecting popular topics does not always increase the likelihood of success due to increased competition. Wider applications of marketing could enhance the ability of environmental crowdfunding campaigns to raise funds.

**Supplementary Information:**

The online version contains supplementary material available at 10.1007/s13280-021-01522-0.

## Introduction

Funding shortages hamper environmental conservation and management efforts, which results in limited ability to mitigate environmental degradation (Wilson et al. 2016; Gill et al. 2017; Waldron et al. 2017). Thus, conservation practitioners need to understand how to effectively improve their fundraising capacity, and much effort has been placed in exploring financial mechanisms to support conservation such as trust funds (Bonham et al. 2014), nature-based tourism (Kubo et al. 2019), and payment for ecosystem services (Adamowicz et al. 2019).

Donations are one of the most important sources of funding for environmental conservation and management as most non-governmental organisations (NGOs) rely heavily on revenue from public donations (Veríssimo et al. 2018). In this context, the success or failure of a fundraising campaign can often determine whether conservation efforts go ahead. In response, various studies have explored what factors drive donations to conservation charities by using data from surveys (Veríssimo et al. 2017), lab and online experiments (Thomas-Walters and Raihani 2017; Curtin and Papworth 2020), field experiments (Kubo et al. 2018), and actual donation campaigns (Veríssimo et al. 2018). However, little is known about online charitable donations for conservation, a recent but increasingly key part of charitable giving (Lundberg et al. 2019). Thus, findings relying on only offline donation behaviours are not sufficient for practitioners to design conservation finance, as in other fundraising fields (e.g. Saxton and Wang 2014).

Online crowdfunding is a new fundraising mechanism that aims to use the wide reach of the internet to collect, usually small, donations from a large number of individuals (Mollick 2014; Sauermann et al. 2019). Many crowdfunding campaigns have been implemented to address environmental issues; however, few researchers have explored crowdfunding in environmental conservation and management (Gallo-Cajiao et al. 2018). One exception was Gallo-Cajiao et al. (2018) which found about 600 conservation-focussed projects since 2009, across 80 countries and corresponding to about 5 million USD in donations. However, this research focussed only on successful crowdfunding projects, as that was the available data, which makes it impossible to understand what differentiates successful and unsuccessful campaigns, which remains an important knowledge gap.

We explore what drives the success of crowdfunding campaigns in environmental conservation and management by using both success and unsuccessful project data from Readyfor (https://readyfor.jp/), a leading Japanese online crowdfunding platform. Data from failure projects are rarely reported (Giakoumi et al. 2018; Godet and Devictor 2018); however, such negative outcome information is essential to enhance future conservation efforts (Game et al. 2014; Catalano et al. 2019; Lenda et al. 2020). By applying a mixed-methods approach including a content analysis and regression analysis, we explore the topical areas that receive attention from environmental crowdfunding campaigns and explore mechanisms driving fundraising success.

## Materials and methods

### The crowdfunding platform

We extracted the publicly available data from the online crowdfunding platform Readyfor, which was launched in March 2011 as Japan's first crowdfunding platform. Projects on Readyfor cover a variety of topics concerning not only environmental conservation and management but also education, art, and technology. The platform includes not only projects based in Japan but also elsewhere in Asia, as well as Europe, North America, and South America, although the website targets mainly Japanese backers. By April 2020, the platform had hosted more than 10 000 projects and raised more than 100 million USD (an estimate based on an exchange rate of 100 Japanese yen (JPY) to the US dollar (USD)). We focussed on projects using the “Environmental Protection” tag attributed by the platform. There are many different project tags used and it should be noted that tags are not exclusive; that is, projects had multiple tags. In terms of fundraising model, Readyfor projects are divided into two categories: All-Or-Nothing and Keep-It-All. In All-Or-Nothing, the campaigner receives the amount donated by backers only if the project meets its fundraising target. In contrast, a Keep-It-All campaigner receives the amount collected by the deadline regardless of the total amount raised. Furthermore, Readyfor provides three campaign types: Reward-base, Charity, and Government. In Reward-base backers obtain rewards according to their contributions. Rewards can include a thank you letter or email, books, local food, or tickets to participate events. Charity projects, also called donation-based projects, are organised by organisations eligible for tax-deductible donation deductions and do not provide any rewards. Government-type projects are organised by local governments, providing tax deductions according to the contributors’ characteristics, such as income. On Readyfor, the duration of the project is set between 10 and 90 days. If the goal (i.e. targeted amount) has been achieved within that period, the campaigner can set additional goals. In this case, the campaigner is able to receive the money collected at the end of the project period even if the total does not reach the new goal.

### Sampling

We extracted the data relative to crowdfunding campaigns in the category of environmental conservation and management created from the 16th of January 2013 to the 26th of September 2019. To better understand crowdfunding success, we extracted relevant characteristics of both successful and unsuccessful projects. We summarise the description of these characteristics in Table 1.

**Table 1:** Variable descriptions

Variable names	Variable type	Descriptions [*Abbreviation* used in the regressions]	Relevant references
*Dependent variables*			
Achievement rate	Numerical	A continuous variable is the ratio calculated by dividing each actual raised amount by each fundraising goal, which describes what extent can be achieved	Frydrych et al. (2014), Mollick (2014), Zheng et al. (2014), Kim et al. (2016),
Project success	Categorical	A dummy variable indicating whether the campaign was successful. The value is one when the Achievement rate exceeds 1, and zero otherwise	Kunz et al. (2017), Anglin et al. (2018)
*Independent variables*			
(Project characteristics)			
Campaign type	Categorical	A categorical variable composes three levels: Reward-based, Charity [*Charity*], and Government [*Government*]	Kuppuswamy and Bayus (2018)
Project model	Categorical	A categorical variable includes two levels: All-Or-Nothing [*AON*] and Keep-It-All [*KIA*]	Cumming et al. (2019)
Reward-type count	Numerical	Number of reward types in each project [*Reward*]	Kunz et al. (2017)
Picture count	Numerical	Number of pictures on each project website [*Picture*]	Kunz et al. (2017), Xu (2018)
Video count	Numerical	Number of videos on each project website [*Video*]	Kunz et al. (2017), Xu (2018)
Word count	Numerical	Number of characters in the body on each project website [*Word*]	Gafni et al. (2018)
(Campaigners’ experience and marketing strategies)			
Experienced campaigners	Categorical	A dummy variable indicating whether a campaigner had experiences of other campaigns. The value is one when a campaigner had run crowdfunding campaigns on Readyfor before, and zero otherwise	Courtney et al. (2016), Hsu (2007)
Facebook share count	Numerical	Number of shares on Facebook in each project [*Facebook*]	Kromidha and Robson (2016)
Tag count	Numerical	Number of tags attributed by the platform (e.g. animal) [*Tag*]	Lundberg et al. (2019)
Announce count	Numerical	Number of announcements by a campaigner in each project to measure updates [*Announce*]	Mollick (2014), Shahab et al. (2019)
Competitor count	Numerical	The maximum number of other projects running simultaneously [*Competitor*]	Meer (2014, 2017), Moy et al. (2018)

We use two different measures of crowdfunding success: (1) Achievement rate and (2) Project success (Table 1). Achievement rate is the ratio calculated by dividing the actual raised amount by a projects target goal, which describes the extent to what the proposed target was achieved (Frydrych et al. 2014; Kim et al. 2016). We defined it as a success when the Achievement rate exceeds 1 (Pitschner and Pitschner-Finn 2014; Hörisch 2015). Project success was defined as a is a binary outcome to distinguish when a project meets its intent fundraising target.

### Analysis

Our analysis consisted of three parts. We first described characteristics of the projects by considering fundraising outcomes: success or failure (Mollick 2014; Pitschner and Pitschner-Finn 2014; Hörisch 2015). The analysis included the extraction of frequent keywords from the project description to better understand the topical focus of projects. Words in Japanese with the same meaning in English were manually aggregated into a single English word in the process of the translation. We then conducted a content analysis of this dataset. Following previous literature (Mitsui et al. 2018; Heberling et al. 2019; Lee et al. 2020), high-frequency nouns were selected as keywords by using the frequency-inverse document frequency (tf-idf) approach (Altaweel et al. 2019). Then, the keywords were summarised by Principal Component Analysis and a k-means cluster analysis was implemented using the scores to classify the projects based on the contents. The frequency of different keywords in each cluster is presented using a word cloud (Fig. 2; Bercht and Wijermans 2019; Anand and Radhakrishna 2020).

We then developed econometric models to obtain insights into what characteristics contribute to fundraising success. By considering the two outcome measures (i.e. dependent variables) separately, we applied two types of models: ordinary least square (OLS) regressions for Achievement rate, and binary logistic regressions for Project success. We selected the independent variables based on previous crowdfunding literature (see Table 1).

First, we included six project characteristics: Campaign type, Project model, Reward-type count, Picture count, Video count, and Word count. Previous literature, for example, found that an All-Or-Nothing model (i.e. no funding if the campaign fails to achieve the fundraising goal) had a higher chance to raise the money (Cumming et al. 2019) while Kunz et al. (2017) revealed that rewards contributed to crowdfunding success. Also, many studies found that the characteristics of project descriptions, such as length, had an influence on fundraising outcomes (Gafni et al. 2018; Lagazio and Querci 2018; Zhou et al. 2018).

Second, we included independent variables related to the experience of campaigners and their marketing strategies. For example, campaigners’ experience has been shown to impact crowdfunding success (Hsu 2007; Courtney et al. 2016). Also, following the literature (Mollick 2014; Zheng et al. 2014; Kromidha and Robson 2016; Kunz et al. 2017), we explored the impacts of the number of the Facebook shares, tags, and the announcements by campaigners on each campaign website to understand the influence of social networks and online findability of crowdfunding projects. We also investigated the effects of the existence of competitors (i.e. the other projects in the same general topic running simultaneously) to gain insight into potential resource constraints of donors. This hoped to reconcile the mixed findings of previous crowdfunding studies on the impact of competition (Meer 2014, 2017; Moy et al. 2018).

In summary, based on the above rationale, we built the following econometric models (See Table 1 for variable descriptions):

1 $${Achievement rate}_{i}^{*}={\beta }_{0}+{\beta }_{1}{Charity}_{i}+{\beta }_{2}{Government}_{i}+{\beta }_{3}{KIA}_{i}+{\beta }_{4}{Reward}_{i}+{\beta }_{5}{Picture}_{i}+{\beta }_{6}{Video}_{i}+{\beta }_{7}{Word}_{i}+{\beta }_{8}{Word}_{i}^{2}+{\beta }_{9}{Landscape}_{i}+{\beta }_{10}{Sustainable}_{i}+{\beta }_{11}{Experience}_{i}+{\beta }_{12}{Facebook}_{i}+{\beta }_{13}{Tag}_{i}+{\beta }_{14}{Announce}_{i}+{\beta }_{15}{Competitor}_{i}+{\varepsilon }_{i},$$

and

2 $${Crowdfunding success}_{i}^{*}={\beta }_{0}+{\beta }_{1}{Charity}_{i}+{\beta }_{2}{Government}_{i}+{\beta }_{3}{KIA}_{i}+{\beta }_{4}{Reward}_{i}+{\beta }_{5}{Picture}_{i}+{\beta }_{6}{Video}_{i}+{\beta }_{7}{Word}_{i}+{\beta }_{8}{Word}_{i}^{2}+{\beta }_{9}{Landscape}_{i}+{\beta }_{10}{Sustainable}_{i}+{\beta }_{11}{Experience}_{i}+{\beta }_{12}{Facebook}_{i}+{\beta }_{13}{Tag}_{i}+{\beta }_{14}{Announce}_{i}+{\beta }_{15}{Competitor}_{i}+{\varepsilon }_{i}.$$

All analyses were conducted using R version 4.0.2. Packages ‘RMeCab’ (Ishida 2020), ‘RcppMeCab’ (Kim and Kudo 2018), ‘tidytext’ (Silge and Robinson 2016), and ‘recipes’ (Kuhn and Wickham 2020) were used for data cleaning and content analysis.

## Results

### Descriptive patterns of conservation crowdfunding projects

We identified 473 closed crowdfunding projects (Table 2). Most projects were categorised into the Reward-based type (97.3%) compared to Charity type (2.1%) and Government type (0.6%). Note that all Reward-based-type projects adopted the All-Or-Nothing model. Of those 473 projects, 295 projects (62.4%) were successful and received 4.2 million USD in total; however, about 363 080 USD associated with the 178 unsuccessful projects was returned to contributors because they were All-Or-Nothing projects (i.e. funds raised had to be returned if the fundraising target was not reached).

**Table 2:** Descriptive statistics of crowdfunding campaigns

Categorical variables	All (n = 473)		Success (n = 295)		Failure (n = 178)	
	Observation (%)		Observation (%)		Observation (%)	
Campaign type: Reward-base	460	(97.3)	282	(95.6)	178	(100.0)
Campaign type: Charity	10	(2.1)	10	(3.4)	0	(0.0)
Campaign type: Government	3	(0.6)	3	(1.0)	0	(0.0)
Project model: All-Or-Nothing	471	(99.6)	293	(99.3)	178	(100.0)
Project model: Keep-It-All	2	(0.4)	2	(0.7)	0	(0.0)
Experienced campaigners	60	(12.7)	46	(15.6)	14	(7.9)

Numerical variables	Mean	S.D	Median	Mean	S.D	Median	Mean	S.D	Median
Achievement rate	0.923	0.774	1.06	1.37	0.633	1.17	0.185	0.232	0.0985
Reward types count	6.96	3.39	6	7.56	3.53	7	5.97	2.91	5
Picture count	10.4	6.48	9	10.7	5.82	10	9.87	7.43	8
Video count	0.211	0.827	0	0.241	0.865	0	0.163	0.76	0
Word count (*1000)	3.18	1.41	2.84	3.45	1.44	3.19	2.75	1.24	2.49
Facebook share count	77.3	117	34	109	135	60	25.1	42.5	8
Tag count	5.69	2.1	5	6.01	2.2	6	5.16	1.81	4
Announce count	8.75	21.9	5	10.6	9.95	8	5.72	33.2	1
Competitor count	27.7	13.6	26	27.6	13	26	27.8	14.5	26

**Fig. 1:**
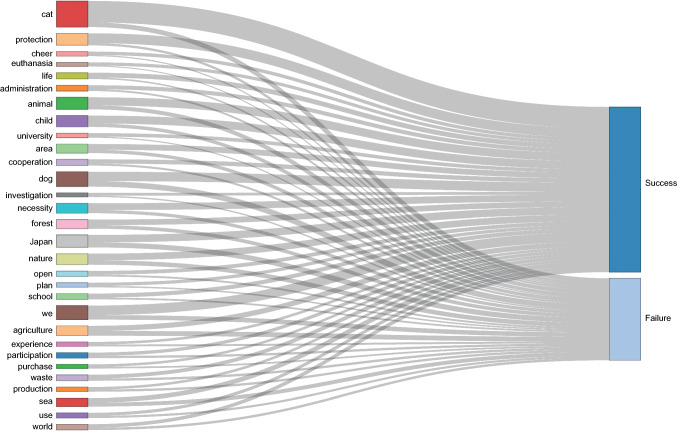
Sankey diagram quantifying Top 30 keywords associated with successful and unsuccessful crowdfunding campaigns

**Fig. 2:**
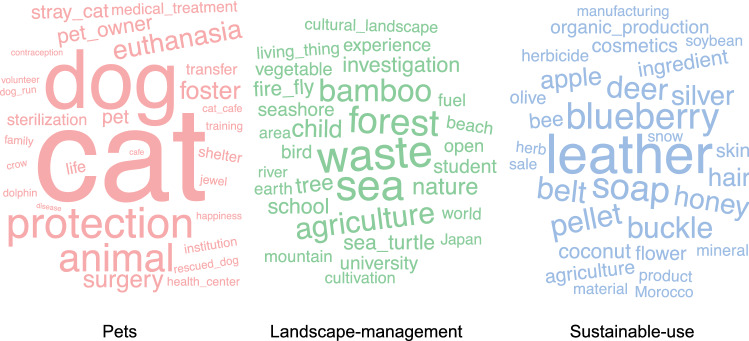
Word clouds composed of Top 30 high frequent keywords, divided by cluster: Pets, Landscape management, and Sustainable use. The detailed frequencies are reported in Table S1 in Supplementary Materials

We then identified the words that most frequently appeared on the descriptions of successful and unsuccessful projects (Fig. 1). By applying a cluster analysis using these keywords, we found three clusters (Fig. 2) which we labelled as Pets (n = 155), Landscape-management (n = 272), and Sustainable-use (n = 46). Projects in the Pets cluster aimed to enhance domestic animal care, including feral cat and dog adoptions, and development of animal shelters and pet cafés (see Plourde (2014) for details on cat cafes for example). Landscape-management projects aimed to protect forest, marine, and coastal landscapes for wildlife conservation and relevant cultures. Sustainable-use projects aspired to develop new products, for example, the skins of deer culled due to human–deer conflicts.

**Fig. 3:**
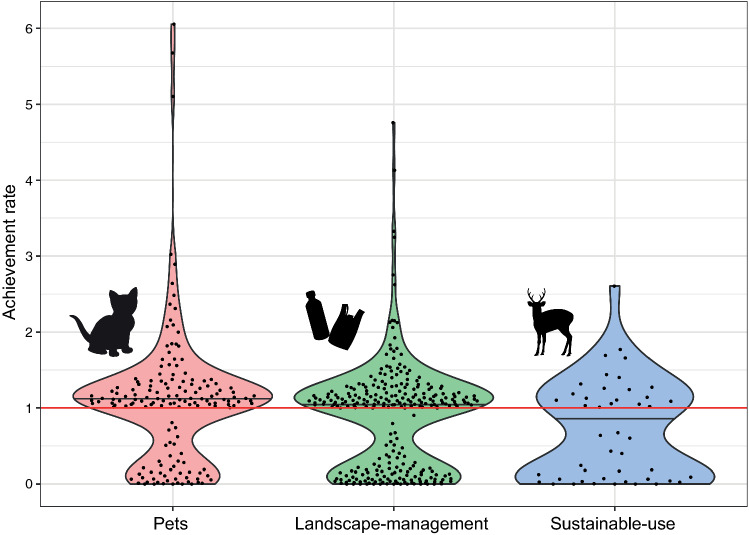
Achievement rate by cluster: Pets, Landscape management, and Sustainable use. The mean achievement rates were 1.08, 0.872, and 0.688, respectively

We compared the mean achievement rates by cluster (Fig. 3). The highest achievement rate cluster was Pets (1.08; SD = 0.902), followed by Landscape management (0.872; SD = 0.696), and Sustainable use (0.688; SD = 0.649).

### What characteristics are associated with successful fundraising?

Regression analysis showed that all parameters’ signs were consistent between the models addressing the Achievement rate and the Project success, and variables associated with successful projects were similar (Table 3).

**Table 3:** Coefficients for estimated models on Achievement rate and Project success

	Achievement rate (OLS)			Project success (Logit)		
	Coefficients		Std. Error	Coefficients		Std. Error
Campaign type: Charity	0.694	***	0.232	15.820		806.949
Campaign type: Government	0.444		0.452	14.672		1141.404
Project model: Keep-It-All	− 0.504		0.554	− 0.404		1586.864
Reward-type count	0.026	**	0.011	0.040		0.044
Picture count	− 0.012	**	0.006	− 0.049	**	0.024
Video count	0.041		0.041	0.079		0.148
Word count (*1000)	0.402	***	0.092	0.960	***	0.324
Square of Word count	− 0.036	***	0.011	− 0.080	**	0.037
Cluster (Landscape-management)	− 0.288	***	0.074	− 0.683	***	0.262
Cluster (Sustainable-use)	− 0.438	***	0.125	− 1.009	**	0.427
Experienced campaigners	0.260	***	0.097	0.432		0.384
Facebook share count	0.001	***	0.0003	0.018	***	0.003
Tag count	0.025		0.017	0.158	**	0.064
Announce count	− 0.0001		0.001	0.004		0.005
Competitor count	− 0.004	*	0.002	− 0.014	*	0.009
Constant	0.043		0.185	− 2.276	***	0.658
Observations	473			473		
*R*^2^	0.233					
Adjusted *R*^2^	0.207					
Log Likelihood				− 233.37		
Akaike Inf. Crit				498.741		
Residual Std. Error	0.689		(df = 457)			
*F* Statistic	9.23	***	(df = 15; 457)			

*p < 0.1; **p <  0.05; ***p < 0.01

Concerning project characteristics, charity-type projects and more campaign rewards were positively related to project success while more pictures were negatively related. Note that the coefficient concerning the charity type was only statistically significant for the Achievement rate, and the coefficient concerning the reward was only significant for the Project success. We also found an inverted-U curve regarding the influence of word count on project success, with very long and very short project descriptions being associated with unsuccessful campaigns. We estimate that about 2900 words is the length most closely associated with project success. Furthermore, projects in the Pets cluster tended to be more successful.

Concerning the external variables, experienced campaigners, more Facebook shares and a larger number of tags were positively related to the crowdfunding success (although the latter was not associated with Achievement rate). We also found that the existence of competitors was associated with lower likelihood of success.

As a robustness check, we also conducted sensitivity analyses to ensure the robustness of our results. In particular, we conducted analyses using only “Reward-based” data in addition to the application of other model forms since there were limited samples associated with Charity and Government types and the Keep-It-All model. Results showed that the parameter signs and effects were virtually the same as our results described in Table 3; see Table S2 in Supplementary Materials for details.

## Discussion

Online crowdfunding can help fill the funding gap affecting environmental conservation and management (Gallo-Cajiao et al. 2018). However, focussing only on successful crowdfunding projects can obscure the fact that many projects fail to achieve their targets and thus underestimate the true costs of crowdfunding. The present study used data from both successful and unsuccessful crowdfunding projects to uncover that some project characteristics (e.g. rewards), as well as campaigner experience, promotional effort through social networks, and online findability are associated with crowdfunding success, whereas more pictures and the existence of competitors were associated with project failure.

The present study illustrated that crowdfunding projects using the tag “Environmental Protection” covered a variety of topics across environmental conservation and management. This diversity implied a potential for crowdfunding to contribute towards several conservation issues associated with pet, landscape, and wildlife management. Our results demonstrate that high-frequency keywords are related not only to the project success but also to failure (Fig. 1), which supports the argument that looking only at data from successful cases can be misleading (Game et al. 2014; Catalano et al. 2019). For example, most efforts associated with “agriculture” projects were in vain because a half of the projects including the keywords were unsuccessful. Our findings suggest that it is key to use data from both successful and unsuccessful projects to get a reliable picture of the traits that are associated with higher probability of success.

The Pets cluster projects had the highest mean achievement rate, which suggests domestic animals elicit more support than wild ones (Fig. 2). This supports previous findings that suggest people tend to contribute to more familiar topics (Lundberg et al. 2019) although findings in this area have been mixed (Veríssimo et al. 2018). It is worth noting that the management of pets has attracted considerable controversy and is not universally seen as an environmental conservation (McMillan et al. 2020). However, we believe that for example, the management of feral cats and dogs is a highly pertinent issue within the context of managing risk from invasive species, and environmental conservation and management more broadly (Medina et al. 2011; Hughes and Macdonald 2013). Domestic animals such as cats and dogs remain substantial threats to wildlife (Lepczyk et al. 2004; Longcore et al. 2009; Loss and Marra 2017), and projects aiming to remove them from the wild can be an important management strategy (Lohr and Lepczyk 2014; Mameno et al. 2017). This link, alongside the ability of these animals to capture donations means there is a potential opportunity to be explored by conservationists, although further research is needed to explore the complex interplay between pet ownership and interest in biodiversity conservation (Crowley et al. 2020).

Our findings concerning the negative impact of competitors (i.e. other campaigns on the same broad topic being running simultaneously) implied for example that the greater attention received by domestic animals, reduces the public support received by other environmental conservation projects. More generally speaking, our results suggest that since budgets are limited not only for practitioners but also for backers, an increase in the number of campaigns means that backers can support only a fraction of the campaigns they would otherwise support, or that each campaign will receive less per backer, in any case reducing the likelihoods of any given campaign to meet their fundraising targets. Our results suggest thus that selecting the topic that is preferred by most donors may not lead to a greater chance of success and those hoping to launch a fundraiser may be better from focussing on topics for which there is less competition. This result is not surprising, as for example social marketers have long recognised competition as one key aspect to be considered when designing a behaviour change intervention (Andreasen 2002). While the issue of competition between fundraising initiatives had already been raised in the context of NGO fundraising conducted using conservation flagship species (Veríssimo et al. 2011), there was limited empirical support for it thus far (Veríssimo et al. 2017).

Consistent with the findings of previous research, we also found that there were few campaigns that ended close to achieving their goals and most unsuccessful campaigns received few contributions, which implied there was a threshold effect to achieve a targeted goal (Fig. 3). This result is consistent with previous crowdfunding literature (Mollick 2014; Cumming et al. 2019), which suggests that initial contributions and marketing efforts play essential roles for fundraising success. For example, publicly announcing seed money information can increase the likelihood of crowdfunding success as with previous charitable giving research (Landry et al. 2006; Kubo et al. 2018). It also highlighted the importance of effective marketing through crowdfunding platforms like other fundraising measures (Wright et al. 2015; Veríssimo et al. 2017).

Based on our findings, we see that other project characteristics and marketing strategies are also key to increase the likelihood of success. In terms of project characteristics, we found there was an optimal length of project descriptions while an increase in the number of pictures was associated with a reduced likelihood of success (Kunz et al. 2017). The latter findings suggest an excessive use of pictures can negatively influence readability. Further research on the role of images is needed since images have been argued to play a substantial role in shaping perceptions of wildlife and nature more broadly but there has been limited research into their role (Thomas-Walters et al. 2020). We found that a greater diversity of rewards contributed to project success. This follows previous research (Kunz et al. 2017), and suggests that the development of a diversified reward offer is a worthy investment for conservation practitioners investing in crowdfunding. In terms of marketing strategies, we showed that the ease of finding a project also played an important role, with more tags contributing to fundraising success. We also found that Facebook shares contributed to project success (Mollick 2014; Kromidha and Robson 2016), and recommend campaigners invest in enhancing campaign visibility using social media. Support from influencers with large networks could therefore be an important factor. Furthermore, our results support the notion that campaigns registered as charities (i.e. donation-based campaigns) were more successful, which suggests individual campaigners should explore partnerships with NGOs.

Although this study is one of the first studies to explore crowdfunding success and failure in environmental conservation and management, there are a few limitations. First, we used data from a crowdfunding platform based in Japan, as data from both successful and unsuccessful projects were available. However, online crowdfunding is global (Brüntje and Gajda 2016; Sorenson et al. 2016) and further data collection and analysis are required in other countries to understand the extent to which our findings are generalizable. Second, we used keyword frequencies to characterise and group different crowdfunding projects, given the large amount of text involved. Nonetheless, we are aware that this quantitative approach may miss some contextual elements that a qualitative approach would be able to disentangle, and future research should explore the use of more qualitative methodologies to characterise crowdfunding projects. Finally, this study did not explore the actual outcomes of each project on the ground. Paying attention to environmental outcomes and spillover effects is essential (Pfaff and Robalino 2017) if the goal is to conserve the natural environment, although it should be recognised that the global nature of the projects supported makes this additional step a major challenge.

## Conclusion

The present study examined what drives the success of crowdfunding campaigns in environmental conservation by applying a data-driven mixed methods approach to both success and failure project data. Online crowdfunding has substantial potential to address the lack of funding in environmental conservation and management, having the ability to plug funding gaps. Yet, research on conservation online crowdfunding is still nascent. As the demand for funding and the interest in business and marketing applications in the conservation context increase (Veríssimo 2019; Smith et al. 2020), crowdfunding is likely to become more widely used and thus play a growing role in sustaining environmental conservation efforts globally.

## Supplementary Information

Below is the link to the Supplementary Information.

Supplementary Information 1 (PDF 135 kb)
