# Supplementary material for: What determines the success and failure of environmental crowdfunding?
Source: Ambio. 2021 Mar 23;50(9):1659–69. doi: 10.1007/s13280-021-01522-0 (PMC8285433; doi:10.1007/s13280-021-01522-0)
Supplement: Supplementary file 1 — Supplementary Information 1 (PDF 135 kb) [file 13280_2021_1522_MOESM1_ESM.pdf]

Ambio

Electronic Supplementary Materials (Table S1, Table S2)

Title：
What determines the success and failure of environmental crowdfunding?

Authors:
Takahiro Kubo, Diogo Veríssimo, Shinya Uryu, Taro Mieno, Douglas MacMillan

Table S1. Lists of the keywords and their frequencies by campaign outcome (success/failure), which relate to Figure 1. The table also reports the number of projects having each keyword by campaign outcome.

Word	Word freq. (n)	Word freq in successful (n)	Word freq in unsuccessful (n)	Projects (n)	Successful projects (n)	Unsuccessful projects (n)
cat	2513	2067	446	152	115	37
protection	1146	926	220	151	111	40
cheer	433	324	109	214	152	62
euthanasia	395	301	94	86	67	19
life	602	459	143	179	132	47
administration	557	383	174	232	149	83
animal	1162	781	381	186	125	61
child	1087	760	327	300	184	116
university	443	336	107	151	109	42
area	871	549	322	248	153	95
cooperation	599	407	192	293	192	101
dog	1427	895	532	145	92	53
investigation	394	266	128	77	52	25
necessity	957	654	303	336	215	121
forest	877	537	340	145	90	55
Japan	1201	713	488	310	180	130
nature	1061	637	424	239	151	88
open	532	356	176	166	112	54
plan	441	304	137	235	162	73
school	554	387	167	144	91	53
we	1348	918	430	316	210	106
agriculture	948	493	455	158	93	65
experience	427	244	183	125	76	49
participation	525	311	214	180	112	68
purchase	397	236	161	171	101	70
waste	578	355	223	69	41	28
production	454	239	215	144	77	67
sea	812	440	372	113	71	42
use	493	289	204	208	130	78
world	563	333	230	199	124	75

Table S2. Sensitivity analysis by the OLS and the Logit [Reward-based sample model and Additional quadratic variable model]

	Achievement rate (OLS) [Reward-based sample]			Project success (Logit) [Reward-based sample]			Achievement rate (OLS) [Additional quadratic variables]			Project success (Logit) [Additional quadratic variables]		
	Coefficients		Std. Error	Coefficients		Std. Error	Coefficients		Std. Error	Coefficients		Std. Error
Campaign type: Charity	—	—	—	—	—	—	0.691	***	0.233	15.848		806.419
Campaign type: Government	—	—	—	—	—	—	0.444		0.453	14.634		1136.788
Project model: Keep-It-All	—	—	—	—	—	—	-0.497		0.555	-0.428		1584.087
Reward-type count	0.025	**	0.012	0.040		0.044	0.026	**	0.012	0.042		0.045
Picture count	-0.012	**	0.006	-0.049	**	0.024	-0.011		0.012	0.025		0.072
Square of Picture count	—	—	—	—	—	—	-0.00002		0.0003	-0.003		0.002
Video count	0.038		0.042	0.079		0.148	0.017		0.083	0.094		0.327
Square of Video count	—	—	—	—	—	—	0.004		0.011	-0.003		0.039
Word count (*1000)	0.403	***	0.092	0.960	***	0.324	0.401	***	0.094	0.839	**	0.343
Square of Word count	-0.036	***	0.011	-0.080	**	0.037	-0.036	***	0.011	-0.067	*	0.039
Cluster (Landscape-management)	-0.287	***	0.074	-0.683	***	0.262	-0.286	***	0.075	-0.698	***	0.264
Cluster (Sustainable-use)	-0.435	***	0.125	-1.009	**	0.427	-0.439	***	0.125	-1.055	**	0.431
Experienced campaigners	0.260	***	0.098	0.432		0.384	0.260	***	0.098	0.418		0.384
Facebook share count	0.001	***	0.0003	0.018	***	0.003	0.001	***	0.0003	0.017	***	0.003
Tag count	0.025		0.017	0.158	**	0.064	0.026		0.017	0.157	**	0.064
Announce count	-0.0001		0.001	0.004		0.005	-0.0001		0.001	0.003		0.005
Competitor count	-0.004	*	0.002	-0.014	*	0.009	-0.004	*	0.002	-0.014	*	0.009
Constant	0.048		0.185	-2.276	***	0.658	0.038		0.188	-2.442	***	0.677
Observations	460			460			473			473		
R2	0.216						0.233					
Adjusted R2	0.195						0.204					
Log Likelihood				-233.370						-232.702		
Akaike Inf. Crit.				492.741						501.404		
Residual Std. Error	0.690		(df = 447)				0.691		(df = 455)			
F Statistic	10.256	***	(df = 12; 447)				8.117	***	(df = 17; 455)			

* $p < 0.1$; ** $p < 0.05$; *** $p < 0.01$
